# Automatic segmentation of human knee anatomy by a convolutional neural network applying a 3D MRI protocol

**DOI:** 10.1186/s12891-023-06153-y

**Published:** 2023-01-18

**Authors:** Carl Petter Skaar Kulseng, Varatharajan Nainamalai, Endre Grøvik, Jonn-Terje Geitung, Asbjørn Årøen, Kjell-Inge Gjesdal

**Affiliations:** 1 Sunnmøre MR-klinikk, Langelandsvegen 15, Ålesund, 6010 Norway; 2 Norwegian University of Science and Technology, Larsgaardvegen 2, Ålesund, 6025 Norway; 3 Norwegian University of Science and Technology, Høgskoleringen 5, Trondheim, 7491 Norway; 4 Møre og Romsdal Hospital Trust, Postboks 1600, Ålesund, 6025 Norway; 5 Faculty of Medicine, University of Oslo, Klaus Torgårds vei 3, Oslo, 0372 Norway; 6 Department of Radiology, Akershus University Hospital, Postboks 1000, Lørenskog, 1478 Norway; 7 Department of Orthopedic Surgery, Institute of Clinical Medicine, Akershus University Hospital, Problemveien 7, Oslo, 0315 Norway; 8 Oslo Sports Trauma Research Center, Norwegian School of Sport Sciences, Postboks 4014 Ullevål Stadion, Oslo, 0806 Norway

**Keywords:** Magnetic Resonance Imaging, Musculoskeletal, Deep learning, Knee images segmentation, Visualization

## Abstract

**Background:**

To study deep learning segmentation of knee anatomy with 13 anatomical classes by using a magnetic resonance (MR) protocol of four three-dimensional (3D) pulse sequences, and evaluate possible clinical usefulness.

**Methods:**

The sample selection involved 40 healthy right knee volumes from adult participants. Further, a recently injured single left knee with previous known ACL reconstruction was included as a test subject. The MR protocol consisted of the following 3D pulse sequences: T1 TSE, PD TSE, PD FS TSE, and Angio GE. The DenseVNet neural network was considered for these experiments. Five input combinations of sequences (i) T1, (ii) T1 and FS, (iii) PD and FS, (iv) T1, PD, and FS and (v) T1, PD, FS and Angio were trained using the deep learning algorithm. The Dice similarity coefficient (DSC), Jaccard index and Hausdorff were used to compare the performance of the networks.

**Results:**

Combining all sequences collectively performed significantly better than other alternatives. The following DSCs (±standard deviation) were obtained for the test dataset: Bone medulla 0.997 (±0.002), PCL 0.973 (±0.015), ACL 0.964 (±0.022), muscle 0.998 (±0.001), cartilage 0.966 (±0.018), bone cortex 0.980 (±0.010), arteries 0.943 (±0.038), collateral ligaments 0.919 (± 0.069), tendons 0.982 (±0.005), meniscus 0.955 (±0.032), adipose tissue 0.998 (±0.001), veins 0.980 (±0.010) and nerves 0.921 (±0.071). The deep learning network correctly identified the anterior cruciate ligament (ACL) tear of the left knee, thus indicating a future aid to orthopaedics.

**Conclusions:**

The convolutional neural network proves highly capable of correctly labeling all anatomical structures of the knee joint when applied to 3D MR sequences. We have demonstrated that this deep learning model is capable of automatized segmentation that may give 3D models and discover pathology. Both useful for a preoperative evaluation.

**Supplementary Information:**

The online version contains supplementary material available at 10.1186/s12891-023-06153-y.

## Background

Digital image segmentation involves the labeling of each pixel or voxel into different regions which exhibit the same set of attributes. When applied to medical images, these segmentations may support surgical planning, promote patient empowerment, aid students in education through augmented or virtual reality visualization, provide input for three-dimensional (3D) printing and be the initial step to achieve surgical simulators using personal data [1–3]. Furthermore, it may be implemented as a tool for diagnostic interpretation, allowing precise volume estimation and tissue localization in three spatial dimensions [4–6].

The diagnosis of knee injuries relies on a summary of the information collected through injury history, imaging and clinical examination. However, the most specific knee instability tests require numerous repetitions and training in order to achieve the skills warranted and there are numerous examples in the clinical world that some of these knee injuries are diagnosed years after the original knee incident [7]. Recent research has identified artificial intelligence (AI) as a tool to predict the need for overnight hospital stays in ligament surgery and total knee arthroplasty (TKA) [8]. AI has also contributed to new advanced treatments of meniscus injuries [9]. Orthopedic surgery has made significant progress by adapting new techniques as arthroscopy in the last century and machine learning possess another potential for a ground breaking shift towards optimal treatment. The first step is to delineate normal anatomy and explore the potential of this tool to reveal knee pathology in order to initiate therapeutic handling as early as possible.

Most commonly, the annotation of MR images involves manual labeling of gray scale image data. Although established semi-automated methods such as region-growing, intensity thresholding, and logical operators contribute to manual annotation efficiency, it is time-consuming and labor expensive. With the advancement of artificial intelligence and machine learning methods, the possibility of rapidly yielding accurate automatic segmentations of medical images is introduced [4, 10]. In radiology, convolutional neural network (CNN) algorithms have proven to be a technique ideally suited for image-based tasks such as segmentation, object detection, classification, and image generation, among others [10–13].

The performance of CNNs is not only related to the algorithms themselves but also depends on the availability of image features and contextual information in the datasets applied [14, 15]. The quality and quantity of the grayscale images and of the manually annotated ground truth definitions are therefore strongly related [16, 17]. The majority of studies regarding deep learning segmentation of joints are based on datasets comprising protocols of either single or multiple channels of two-dimensional (2D) MR sequences [18–23]. The slice thickness in MRI is usually in the range of 1 - 3 mm. In addition, most datasets possess a limited number of anatomical classes defined in the ground truth, often narrowed down to bone, meniscus, and cartilage. Segmenting multiple tissues is important for a more complete anatomical visualization [20, 24]. For virtual and augmented surgical simulators, the benefit of a complete knee segmentation furnishes realism and may aid in navigating anatomical landmarks [25–29]. Segmenting all the different tissues is often a demanding task due to morphological complexity, homogeneous intensities and class imbalance of data [30].

The rendering of an anisotropic multislice 2D MR scan has the disadvantage of a decreased through-plane resolution [31]. For a more realistic spatial representation of all anatomical structures in three dimensions, it is recommended to operate with isotropic voxels of reasonably high resolutions [32, 33]. The hypothesis is that, in relation to neural networks, implementing a protocol consisting of multiple MRI weightings exhibiting superior resolution will leverage the capacity of image features, leading to better results with more precise 3D models [1, 4, 10, 14, 15, 34]. To further extend the contextual data availability, the ground truth extraction ought to be as complete as possible.

The main purpose of this work was to determine the performance of a convolutional neural network as a deep learning method to automatically segment musculoskeletal anatomy of the human knee for visualization. The ultimate aim will be to use these models to make 3D models for preoperative planning, and use the model to detect pathology.

## Materials and Methods

### Magnetic Resonance Imaging

The study design involved retrospective interpretation of prospectively acquired data. Imaging was performed on a 1.5 Tesla whole-body MR system Philips Achieva software release 3 (Philips), fitted with maximum strength gradients of 33 mT/m and a maximum slew rate of 160 T m$^{-1}$ s$^{-1}$ (Nova gradients). A dedicated eight-channel knee coil was applied. The MR imaging protocol consists of four pulse sequences: T1 TSE, PD TSE, PD FS TSE and Angio GE, and all sequences following a 3D sampling pattern. In comparison with the standard clinical knee protocol applied daily at the imaging center, the segmentation protocol acquires four times the data points (voxels) per examination. The essential imaging parameters of the sequences are presented in Table 1.

**Table 1:** MR protocol imaging parameters. PD = Proton Density, FOV = Field of View, TSE = Turbo Spin Echo, TR = Repetition Time, TE = Echo Time, NSA = Number of Signal Averages, Refoc. ctrl = Refocusing control

Parameter	T1 TSE	PD TSE	PD FS TSE	Angio GE [25]
Scan mode	3D	3D	3D	3D
Imaging plane	Sagittal	Sagittal	Sagittal	Transversal
FOV	160 mm in 3D	160 mm in 3D	160 mm in 3D	160 mm in 3D
Acquisition voxel size	0.5$\times$0.6$\times$0.4	0.5$\times$0.6$\times$0.4	0.5$\times$0.6$\times$0.4	0.8$\times$0.8$\times$0.4
Recon voxel size	0.4$\times$0.4$\times$0.4	0.4$\times$0.4$\times$0.4	0.4$\times$0.4$\times$0.4	0.4$\times$0.4$\times$0.4
Slices	400	400	400	400
Sense factor	2.5 (AP), 2 (RL)	2.5 (AP), 2 (RL)	2.5 (AP), 2 (RL)	4 (AP)
TSE factor	30	100	100	NA
TFE factor	NA	NA	NA	256
Shot interval	NA	NA	NA	3233 ms
Profile order	NA	NA	NA	Low-high
Turbo direction	NA	NA	NA	Radial
Flip angle	60 refoc. ctrl	60 refoc. ctrl	60 refoc. ctrl	90
TR	600 ms	3000 ms	3000 ms	5.4 ms
TE	30 ms	30 ms	30 ms	2.7 ms
NSA	3	2	2	3
Fat suppression	No	No	SPAIR	SPIR
Scan time	10:10 min	10:09 min	10:09 min	06:06 min

**Fig. 1:**
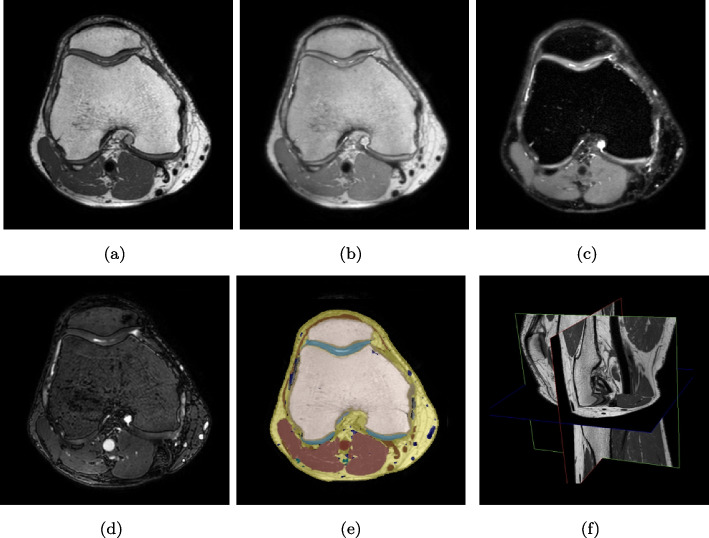
(**a**) Axial T1 TSE, (**b**) Axial PD TSE, (**c**) Axial PD FS TSE (**d**) Axial Angio GE, (**e**) Manually annotated ground truth, (**f**) Volume view of the 3D imaging planes (TSE = Turbo Spin Echo. FS = Fat Saturated)

The gradient echo-based angio sequence [35] was scanned in the transversal plane for maximum inflow effects. This sequence was subsequently reconstructed into 400 slices in the sagittal orientation to match the exact geometrical features of the 3D TSE sequences. This specialized protocol requires approximately 40 minutes compared to the routine examination protocol, which is 15-20 minutes. The imaging sequences T1, PD, FS and Angio are presented as axial projections in Fig. 1.

### Sample selection and dataset

The right knee of 46 participants were scanned exclusively for this study. The sample size was derived from experiences gained empirically during a preliminary pilot study prior to the commencement of this work [36]. An informed and written consent agreement regarding the handling of data was signed by all participants, along with the standard MRI safety sheet. The inclusion criteria were adults with fused growth plates and without any known damage to the knee joint such as fractures, cartilaginous wear, ligamentous tears, and meniscal damage. We excluded two subjects due to excessive movement during the scan. Also, three subjects revealed some apparent pathology and were thus removed. One subject was discarded due to significant wrap-around artifacts.

The included participants were divided into independent subgroups of 20, 5, and 15 for training, validation, and test dataset, respectively. The mean age of the volunteering participants in these subgroups were 36.7, 37.2, and 28.8 years (range: 21 - 75 years) and the ratio of men and women were 13:7, 2:3, and 2:3, respectively. The single pathology case was female age 27. The research was approved by the Norwegian Regional Committee for Medical and Health Research Ethics (REK nr. 61225). The study is performed in accordance with the ethical standards of the 1964 Helsinki declaration and its later amendments or comparable ethical standards.

The DICOM images were initially anonymized and converted to the NIfTI format. For the network to operate correctly, it is imperative that each individual scan has all four input channels co-registered. The ITK-SNAP [37] and NordicICE 4.2.0 (NordicNeuroLab) software packages were used to create the manual ground truth of the 40 healthy participants. The manual segmentations (ground truth) consists of 13 classes including bone medulla, posterior cruciate ligament (PCL) anterior cruciate ligament (ACL), cartilage, meniscus, cortical bone, collateral ligament, tendon, adipose tissue, artery, vein, nerve, and muscle, shown in Fig. 1e. The manual ground truth annotation was performed using selected images from each of the four MR sequences depending on which tissue to annotate, taking advantage of the inherent intensity and contrast characteristics of each sequence. For example the PD FS sequence allowed easier annotation of cartilage, while the Angio sequence was mainly used for vessels. A feature of the annotation software utilized allowed for making a final multiple tissue ground truth by merging the individual tissue ground truths, which in turn reduced the overall manual annotation time. Ground truth annotation of all tissues segmented in this work can be performed using the T1 TSE sequence exclusively, but with considerably longer time spent for the manual annotation. These annotations were crafted by a clinical medical physicist, and validated by a radiologist, both holding over 25 years of experience in their respective fields of work.

### Neural network

**Fig. 2:**
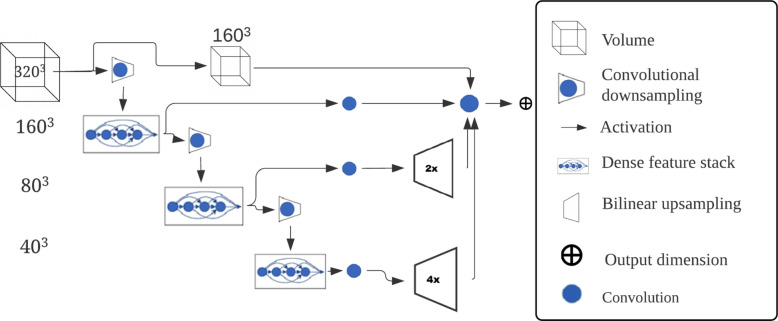
The neural network architecture

Over the recent years, several platforms facilitating the development of deep learning neural networks for medical imaging have emerged from the artificial intelligence community [10, 38]. One of these is NiftyNet [39]-an open-source neural networks platform based on the Tensorflow framework. We have used the DenseVNet convolutional neural network (CNN) for automated knee MR image segmentation, and the network architecture is presented in Fig. 2.

We present the network hyper parameters for training and inference in Table 2. These parameters are detailed in the configuration manual of NiftyNet [40]. The requirement of spatial window size for DenseVNet is set equally divisible by 2 * dilation rates [41]. The dilation rate variable was set to the default value 4, resulting in a mandatory patch size equally divisible by 8. The image patch $320\times 320\times 320$ is taken from each image with batch size one as the input for the network. The image volume is down sampled using convolution with stride 2, and an average pooling to $160\times 160\times 160$ from the input. At the decoder level, there were three dense feature stack (DFS) blocks that outputs a skip connection with a convolution with stride 1, and a convolution with stride 2 for down-sampling the spatial size. Finally, the skip connection channels of the second DFS block and the bottle-neck layer channels were up-scaled to the half spatial size of the input shape. All the output channels (average pooling and skip connections) were concatenated and convoluted to get a single channel output. The Parametric rectified linear unit (PReLU) [42] was chosen as activation function. A combination of dice loss with cross entropy loss (DicePlusXEnt) [43] was applied as the loss function. A constant learning rate [44] of 0.0001 was utilized throughout the training. Data augmentation was achieved through random flipping axes, elastic transformations and random scaling [45]. In order to maximize network quality performance, the patch size was kept as large as possible without exceeding system video memory.

**Table 2:** Selected hyper parameters for training and inference

Parameter	Training	Inference
Window size	320, 320, 320	400, 400, 400
Activation function	PRelu	
Loss type	DicePlusXEnt	
Normalization	Histogram	Histogram
Window sampling	Weighted	Weighted
Volume padding	24, 24, 24	0, 0, 0
Learning rate	0.0001	
Random flipping axes	(0, 2)	
Elastic transformation	True	
Rotation angle	(-10, 10)	
Scaling percentage	(-10, 10)	

The following computer hardware components were acquired for training the network: AMD Ryzen 3900X 12-core CPU, Corsair 128 GB DDR4 memory, and a single NVIDIA TITAN RTX 24GB GPU.

### Validation metrics

The correctness of deep learning segmentation outputs can be measured using the confusion matrix which consists of true positive (TP), false positive (FP), false negative (FN), and true negative (TN). One of the most commonly used metrics is the Dice similarity coefficient (DSC) [46, 47]. DSC measures similarities between the manually segmented ground truth and the automated deep learning predicted segmentation, defined by

$$\begin{aligned} \text {DSC} = \frac{\text {2TP}}{\text {2TP+FP+FN}}. \end{aligned}$$

The Jaccard index is defined as the intersection of AI prediction and the manual segmentation over their union, that is

$$\begin{aligned} \text {Jaccard}= \frac{\text {TP}}{\text {TP} +\text {FP} + \text {FN}}. \end{aligned}$$

Hausdorff distance (HD) measures the distance between the manual segmentation and AI predicted segmentation surfaces. The Hausdorff distance defined by

$$\begin{aligned} \text {HD}(A, B) = \max \left[ {\underset{a \in A}{\sup}}\ {\underset{b \in B}{\inf}}\ d(a,b),\; {\underset{b \in B}{\sup}}\ {\underset{a \in A}{\inf}}\ d(a,b) \right] , \end{aligned}$$

where *A* and *B* are two surfaces, sup and inf represent the supremum and infimum, respectively [48].

We trained the deep learning network with different combinations of image channels to determine the effect of multiple MR weightings on segmentation results. The network was trained for approximately 14 days and terminated at 25,000 iterations for each combination of input channels. The objective of the validation dataset was to determine the optimal iteration in which training should be terminated to avoid network overfitting. The mean Dice score of all label classes were determined for the validation dataset at intervals of one thousand iterations. The iteration corresponding to the highest Dice score for each image channel combination was used to evaluate the test dataset. The results obtained from validating the individual combinations are shown as Supplementary Fig. 1 in the Supplementary materials.

## Results

### Deep learning results

**Fig. 3:**
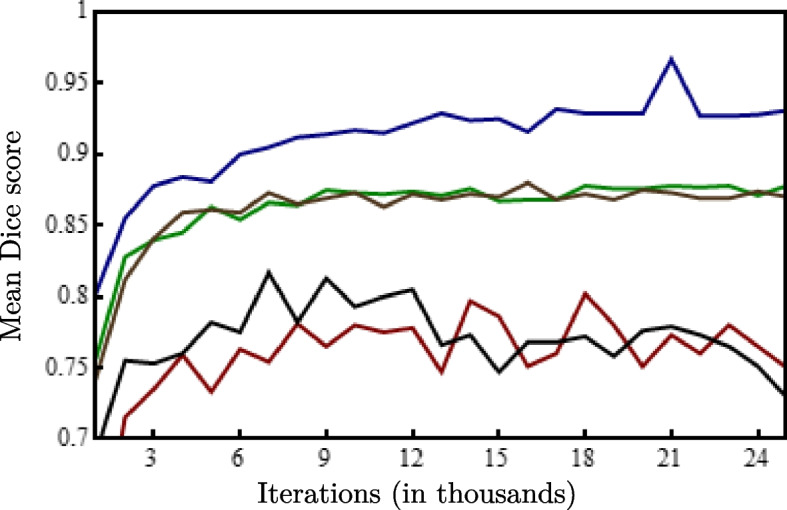
Evaluation of validation dataset by training different combinations of respective image channels (  T1  PDFS,  T1PDFS,  T1FS,  T1PDFSAngio)

Our experiments indicate that the learning of the neural network varies depending on the combination of image channels, shown in Fig. 3. The input image channels T1PDFS and T1FS performs better than the T1 and PDFS sequence combinations. We recognize T1PDFSAngio as the best performing model in comparison with the other combinations of sequences. The T1PDFSAngio model attained optimal learning at 21,000 iterations with the mean Dice score of 0.967 (±0.032) averaged across all 13 classes. The results of the remaining image channel combinations can be found in Supplementary Table 1 of the Supplementary materials.

**Fig. 4:**
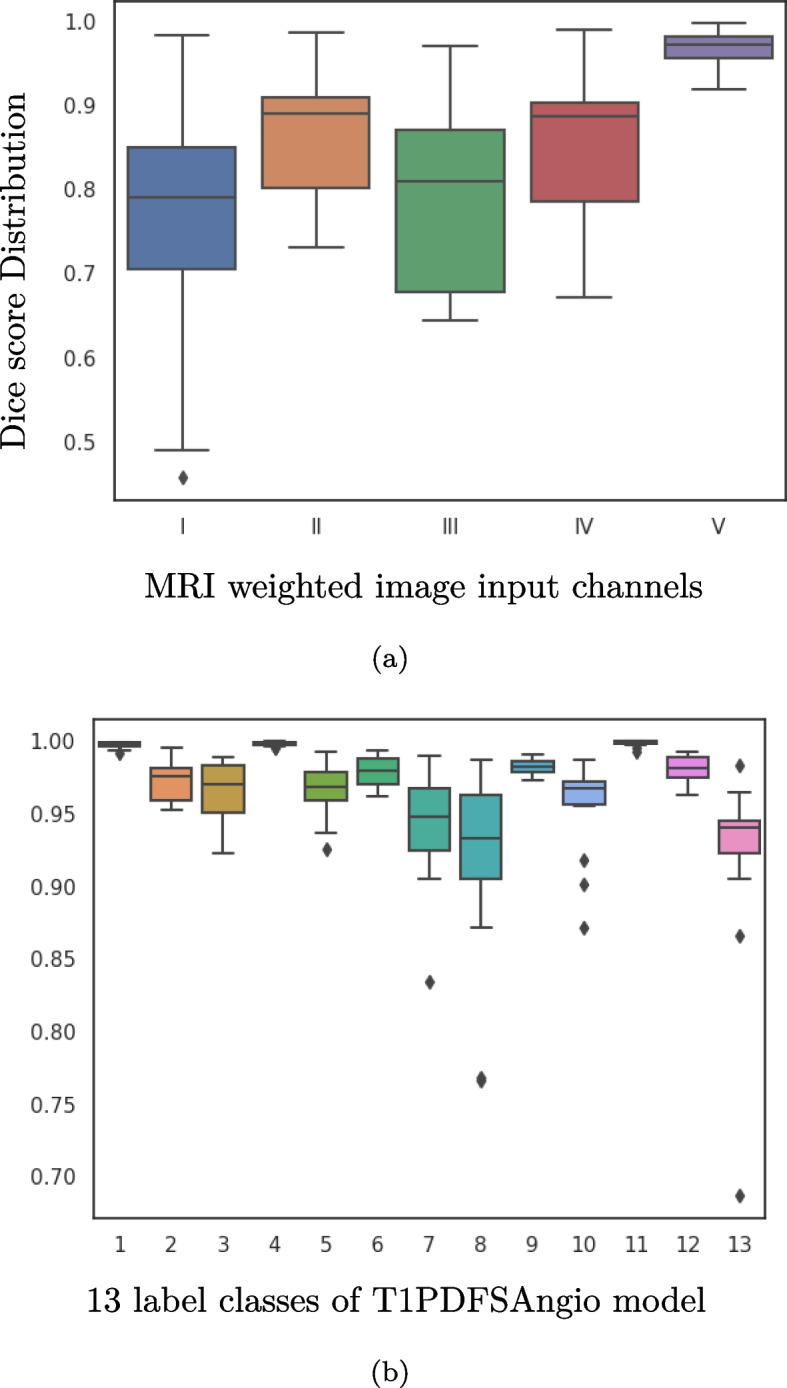
The Dice score evaluation distribution of T1PDFSAngio by 15 subjects of the test dataset (**a**) Different combinations of MRI input data channels of I:T1, II:T1FS, III:PDFS, IV:T1PDFS, V:T1PDFSAngio versus averages of all classes of the test dataset (**b**) the individual anatomical classes 1. Bone medulla, 2. PCL, 3.ACL, 4.Muscle, 5.Cartilage, 6.Cortical bone, 7.Artery, 8.Collateral ligament, 9.Tendon, 10.Meniscus, 11.Adipose tissue, 12.Vein, 13.Nerve

The test dataset was evaluated at the optimal iteration corresponding to the different image channel combination models. We observed that the beneficial effect of combining image channels during validation is reinforced by the test dataset. The outcome is particularly pronounced when training all MR weightings, and the correlation between training different image channels collectively are presented in Fig. 4a. For example, the hypo-intense vascular tissue typically seen in T1 images were ineffective for the network to learn arteries and veins. However, we noticed that adding Angio GE with other sequences to the network elevated the scores of vascular tissue.

The evaluation of test dataset metric for each of the 13 classes is summarized in Fig. 4b and Table 3. The scores are averaged across all classes, resulting in a mean DSC (±standard deviation) of 0.967 (±0.040). The achieved Dice scores were higher and more stable for larger structures such as bone medulla, muscle, and fat, while the scores appeared less for the minor structures. This is believed to be partly due to the class imbalance of the data. Also, the common low intensities of the ligaments, tendons and meniscus might cause these connective tissues to be indistinguishable for the network. Another argument is that some subjects may have more or less degenerative changes to tissues, resulting in false predictions.

**Table 3:** The average Dice scores, Jaccard Index and Haursdorff distances (±standard deviation) of the test dataset evaluated at 21,000 iterations by the neural network model trained with all image channels combined

Class	DSC	Jaccard	Hausdorff Distance
Bone medulla	0.997±0.002	0.994±0.004	42.63±24.79
PCL	0.973±0.015	0.947±0.028	98.30±55.61
ACL	0.964±0.022	0.931±0.042	107.00±71.58
Muscle	0.998±0.001	0.997±0.002	66.94±42.77
Cartilage	0.966±0.018	0.934±0.034	111.90±18.25
Cortical bone	0.980±0.010	0.961±0.019	53.43±28.26
Artery	0.943±0.038	0.894±0.065	68.50±43.88
Collateral ligament	0.919±0.069	0.856±0.109	114.27±39.07
Tendon	0.982±0.005	0.965±0.009	81.40±21.66
Meniscus	0.955±0.032	0.916±0.057	185.60±53.54
Adipose tissue	0.998±0.010	0.997±0.003	16.568±6.685
Vein	0.980±0.010	0.962±0.018	30.36±8.00
Nerve	0.921±0.071	0.860±0.106	114.11±46.61
Average	0.967±0.040	0.940±0.068	83.92±58.15

### Pathology case

**Fig. 5:**
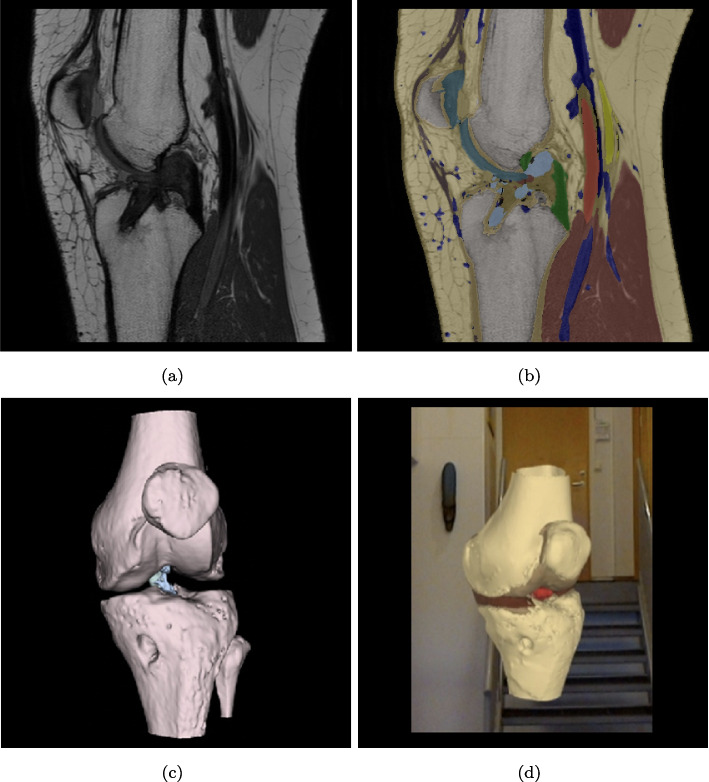
Pathology case: (**a**) Post-surgical proton density fat suppressed TSE (FS TSE) sagittal plane image showing the torn anterior cruciate ligament. (b) Predicted output by the neural network. The tibial drill hole and the intra-articular graft from the reconstructive surgery is visible. (**c**) 3D render of the segmentation. (**d**) Holographic 3D model representation

We chose the trained deep learning network model from the T1PDFSAngio input sequence combination, and inferred the left knee image volume of a post-surgical ACL reconstruction of an adolescent patient. The patient had a complicated displacement of the intra-articular graft preventing full extension, resulting in a mechanically locked knee [49]. The network prediction correctly revealed that the ACL was missing, along with visualization of the tunnel left after drilling the femoral and tibial bone, shown as segmentations and hologram in Fig. 5. The surgical graft visualized in the hologram was annotated manually directly to the neural network inference output.

## Discussion

Generally, neural network performance depends on the algorithms applied and the input data, both quantitatively and qualitatively. In our study, 1600 slices were scanned divided into four pulse sequences per knee. This is considerably more detailed anatomical coverage than most standard clinical protocols. The total protocol scan time used in this study was just shy of 40 min. The scan time reflects that the main objective for this study was detailed anatomic visualization and segmentation based on isotropic data, and not disease detection.

Other relevant research focusing on deep learning of MRI images of the knee joint has been carried out. Most of which are focused on cartilage segmentation and lesion classifications [19–23]. The quantitative evaluation scores obtained in our study cannot be directly compared to the studies listed in Table 4, mainly because different data sets were used. These data sets contain subjects whose images demonstrate pathology which is likely to significantly affect the result. One possible reason for the improved evaluation metrics of the method used in this work could, however, be explained by respecting the fact that the dataset described in the study is based on higher resolution 3D scans of multiple weightings and a more complete annotation of the entire knee anatomy. This reasoning is justified given that both the quality and the quantity of input data affects the performance of this neural network configuration. The very high DSC could also be explained given that the initial starting point for the ground truths were generated using a neural network, and then thoroughly corrected by manually removing false positive voxels and adding voxels where the network had made mistakes. However, because the same ground truths were used to evaluate all different channel combinations, the means to find the best fitting model would not be affected by this approach. The mean DSC calculated across all tissue classes might have a very high DSC owing to the fact that the larger structures such as bone, muscle and adipose tissue will increase the average DSC, even if the smaller structures score lower.

**Table 4:** Other work relevant to automated segmentation of knee structures [19–23]. DSC = Dice Score Coefficient, OAI = Osteoarthritis Initiative, DESS = Double Echo Steady State

Study	Sequences	Subjects	Neural network	Results (DSC ± Standard deviation)	Comment
F. Liu et al. [19]	T2 FS FSE PD FSE T2 Mapping	175	2D encoder-decoder VGG16	Femur (0.96 ± 0.03) Tibia (0.95 ± 0.03) Femoral cartilage (0.81 ± 0.04) Tibial cartilage (0.82 ± 0.04)	
Z. Zhou et al. [20]	PD FS FSE	20	2D encoder-decoder VGG16	Femur (0.970 ± 0.010) Femoral cartilage (0.806 ± 0.062) Tibia (0.962 ± 0.015) Tibial cartilage (0.801 ± 0.052) Patella (0.898 ± 0.033) Patellar cartilage (0.807 ± 0.101) Meniscus (0.831 ± 0.031) Quadriceps and patellar tendon (0.815 ± 0.029) Muscle (0.932 ± 0.024) Joint effusion and Baker’s cyst (0.736 ± 0.069) Infrapatellar fat pad (0.882 ± 0.040) Other non-specified tissues (0.913 ± 0.017)	All subjects had various degrees of Osteoarthritis
A. Tack et al. [21]	DESS	88	3D U-Net	Medial menisci (83.8) Lateral menisci (88.9)	OAI Imorphics dataset
F. Ambellan et al. [22]	DESS	88	3D U-Net	Femoral cartilage (0.89 ± 2.41) Medial tibial cartilage (86.1 ± 5.33) Lateral tibial cartilage (90.4 ± 2.42)	OAI Imorphics dataset
	DESS	507	3D U-Net	Femoral bone (98.5 ± 3.02) Femoral cartilage (89.9 ± 3.25) Tibial bone (98.5 ± 3.25) Tibial cartilage (85.6 ± 4.54)	OAI ZIB dataset
E. Panfilov et al. [23]	DESS	88	U-Net	Femoral cartilage (0.907 ± 0.019) Tibial cartilage (0.897 ± 0.028) Patellar cartilage (0.871 ± 0.046) Meniscus (0.863 ± 0.034)	OAI Imorphics dataset
	DESS	44	U-Net	Femoral cartilage (0.827 ± 0.024) Tibial cartilage (0.816 ± 0.029)	

This study had some noteworthy limitations. The network has been tested using images acquired by a single vendor 1.5T scanner. The training dataset volume is limited to 20 subjects; a larger quantity for training is believed to benefit the neural network in terms of performance. Furthermore, the pathology case was a post-surgical knee and the ability to differentiate between normal anatomy and injured pathology will likely be more sensitive and specific with a broader spectrum of injured knees added to the training data set. External validity may be reduced as is it only tested on a limited number of healthy participants. Evaluation based on matching the predicted output against a validated ground truth is one of the most common methods of evaluating the accuracy of a neural network. However, the method may be debatable as the result is strongly dependent of the validated ground truth definition. A poorly defined validated ground truth consequently causes statistical uncertainty, mainly related to errors of omission and commission. Structures labeled correctly by the neural network might not be defined in the validated ground truth. In other words, a false-positive voxel during validation might be true positive. Differential classification of a given tissue while exerting manual annotation may in some instances be difficult and is prone to individual subjectivity, particularly in anatomical regions occupied by overlapping isointense voxels.

**Fig. 6:**
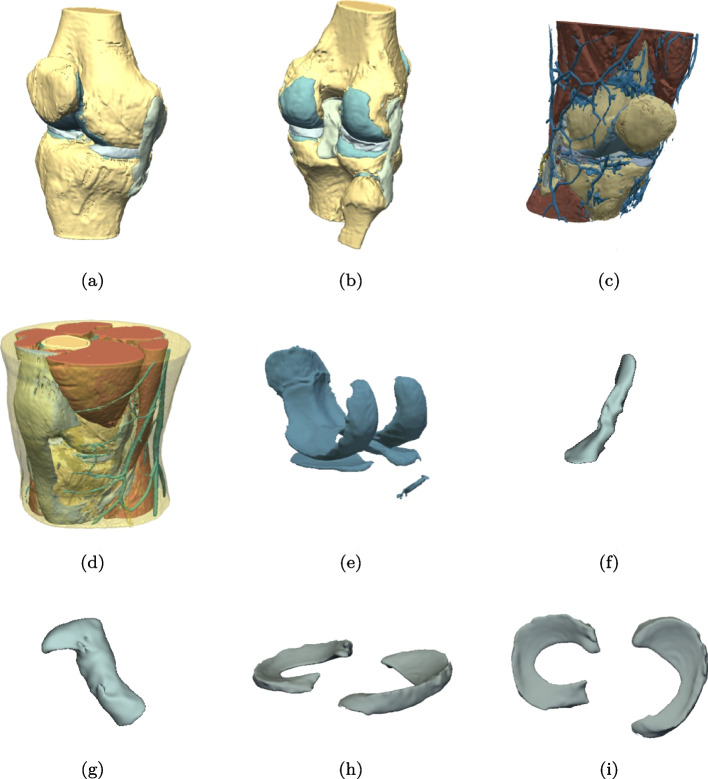
Results of test dataset in 3D (**a**), (**b**) Bone, ACL, PCL, meniscus and collateral ligaments, (**c**) Bone, muscles, ligaments and veins (**d**) Complete segmentation with transparent adipose tissue (**e**) Cartilage, (**f**) ACL, (**g**), PCL, (**h**), (**i**) Meniscus

The obtained spatially realistic segmentations may be of clinical value for surgical planning, patient empowerment, and student education. Moreover, neural network-labeled outputs based on healthy subjects may further be annotated using appropriate software. These extended datasets including labels mimicking pathological lesions may be reintroduced to the neural network and trained progressively. The segmented structures, as shown in Fig. 6, can easily be imported into into a 3D-printing environment. Additionally, they can be made viewable through augmented or virtual reality as inputs to a surgical simulator.

## Conclusion

By introducing an optimized MR imaging protocol for depicting human knee anatomy, our study demonstrates that a convolutional neural network can achieve automatic semantic segmentation with high performance. The prediction of the neural network represents spatially realistic representations of the entire knee joint in three dimensions, which are well suited for visualization purposes.

Figures 5 and 6 are examples of the three-dimensional anatomic outlay of the knee. We may predict that the future will give orthopedists the possibility of having such models available before an operation either 3D printed or as holograms. We also predict that the capability of the model to detect pathology may become a useful tool for both radiologists and orthopedists.

## Supplementary Information

**Additional file 1.** Supplementary materials.

## Data Availability

The datasets generated and/or analysed during the current study are not publicly available due to private interests but are available from the corresponding author on reasonable request.

## Abbreviations

DSC: Dice Similarity Coefficient
CNN: Convolutional Neural Network
MPR: Maximum Projection Reconstruction
TSE: Turbo Spin Echo
FSE: Fast Spin Echo
DESS: Double Echo Steady State
