# Supplementary material for: Automatic segmentation of human knee anatomy by a convolutional neural network applying a 3D MRI protocol
Source: BMC Musculoskelet Disord. 2023 Jan 18;24:41. doi: 10.1186/s12891-023-06153-y (PMC9847207; doi:10.1186/s12891-023-06153-y)
Supplement: Supplementary file 1 — Additional file 1. Supplementary materials. [file 12891_2023_6153_MOESM1_ESM.pdf]

1 Supplementary materials

	T1	T1FS	T1FS	T1PDFS
Bone medulla	0.992 ± 0.001	0.994 ± 0.001	0.939 ± 0.049	0.994 ± 0.001
PCL	0.813 ± 0.058	0.928 ± 0.011	0.795 ± 0.087	0.868 ± 0.020
ACL	0.749 ± 0.111	0.881 ± 0.023	0.804 ± 0.075	0.870 ± 0.029
Muscle	0.939 ± 0.011	0.978 ± 0.001	0.970 ± 0.006	0.982 ± 0.001
Cartilage	0.809 ± 0.028	0.914 ± 0.008	0.855 ± 0.041	0.928 ± 0.004
Bone cortex	0.856 ± 0.010	0.910 ± 0.007	0.776 ± 0.068	0.910 ± 0.009
Artery	0.733 ± 0.034	0.835 ± 0.033	0.751 ± 0.058	0.817 ± 0.034
Collateral ligament	0.654 ± 0.036	0.751 ± 0.052	0.439 ± 0.129	0.675 ± 0.084
Tendon	0.761 ± 0.020	0.887 ± 0.012	0.706 ± 0.059	0.894 ± 0.018
Meniscus	0.805 ± 0.035	0.892 ± 0.021	0.876 ± 0.027	0.907 ± 0.019
Adipose tissue	0.913± 0.025	0.963 ± 0.008	0.919 ± 0.025	0.972 ± 0.006
Vein	0.408 ± 0.022	0.718 ± 0.015	0.665 ± 0.031	0.734 ± 0.016
Nerve	0.618 ± 0.119	0.766 ± 0.054	0.636 ± 0.066	0.804 ± 0.045

Supplementary Table 1: Mean DSC ± standard deviation of the respective image channel combinations for each individual anatomical class at 21,000 iterations

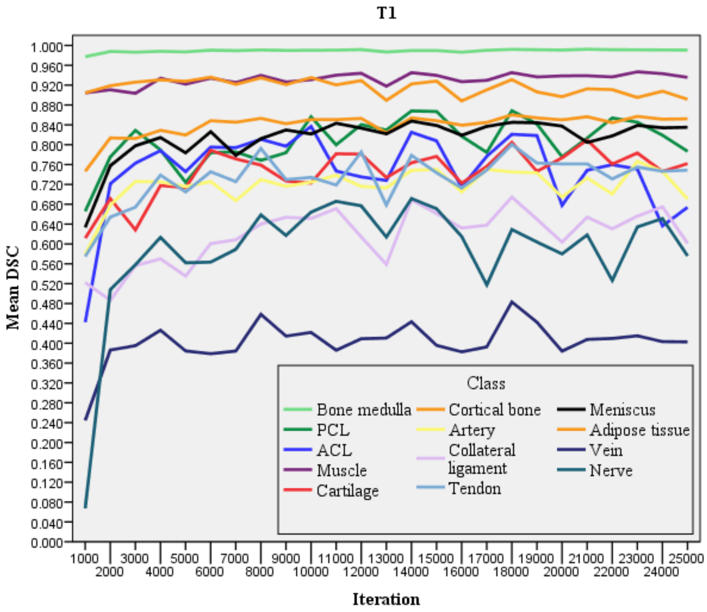

(a) Training T1 alone

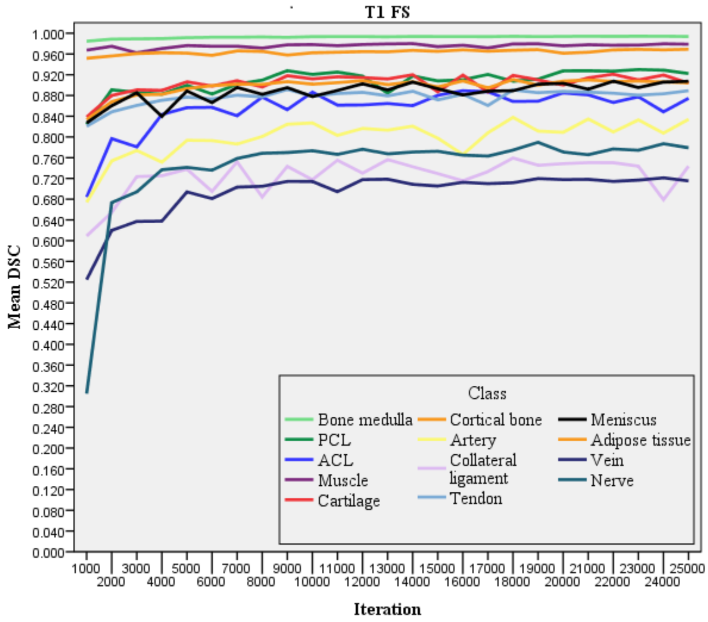

(b) Combining T1 & FS

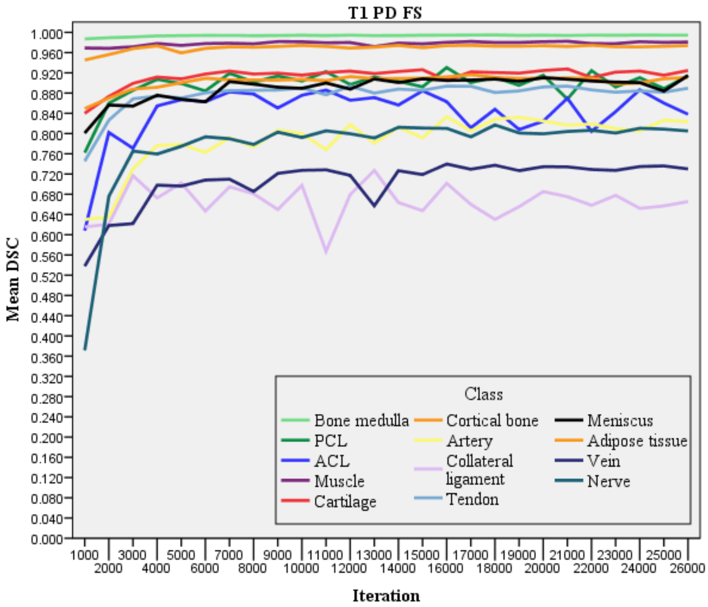

(c) T1, PD & FS combination

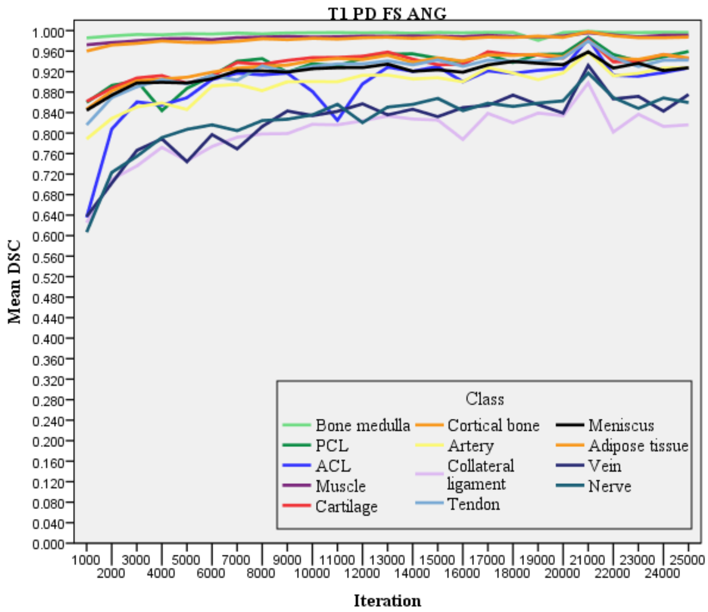

(d) All the channels T1, PD, FS, Angio together.

Supplementary Figure 1: Validation curves of the DSC obtained during training for (a) Training T1 alone, (b) combining T1 & FS (c) T1, PD & FS combination and (d) all the channels T1, PD, FS, Angio together.
